# Safety and pharmacodynamics of a single infusion of danavorexton in adults with idiopathic hypersomnia

**DOI:** 10.1093/sleep/zsad049

**Published:** 2023-03-08

**Authors:** Emmanuel Mignot, Richard K Bogan, Helene Emsellem, Nancy Foldvary-Schaefer, Melissa Naylor, Rachel Neuwirth, Hélène Faessel, Todd Swick, Tina Olsson

**Affiliations:** Stanford Department of Psychiatry and Behavioral Medicine, Center for Sleep Sciences and Medicine, Stanford University Medical School, Palo Alto, CA, USA; Bogan Sleep Consultants, LLC, Columbia, SC, USA; The Center of Sleep & Wake Disorders, Chevy Chase, MD, USA; Department of Neurology, Sleep Disorders Center, Cleveland Clinic, Cleveland, OH, USA; Takeda Development Center Americas, Inc., Lexington, MA, USA; Takeda Development Center Americas, Inc., Lexington, MA, USA; Takeda Development Center Americas, Inc., Lexington, MA, USA; Takeda Development Center Americas, Inc., Lexington, MA, USA; Takeda Development Center Americas, Inc., Lexington, MA, USA

**Keywords:** excessive daytime sleepiness, idiopathic hypersomnia, orexin type 2 receptor agonist, danavorexton

## Abstract

**Study Objectives:**

Idiopathic hypersomnia (IH) is a chronic disorder characterized by excessive daytime sleepiness unexplained by another disorder or drug/medication use. Although the orexin system plays a role in sleep-wake regulation, orexin A levels in the cerebrospinal fluid are normal in people with IH. This phase 1b, randomized, placebo-controlled, crossover study aimed to investigate the safety, pharmacokinetics, and pharmacodynamics of danavorexton, a small-molecule orexin-2 receptor agonist, in adults with IH.

**Methods:**

Adults with IH aged 18–75 years were randomized to one of two treatment sequences of single intravenous infusions of danavorexton 112 mg and placebo. Pharmacodynamic endpoints included the maintenance of wakefulness test (MWT), the Karolinska Sleepiness Scale (KSS), and the psychomotor vigilance task (PVT). Adverse events were monitored throughout the study period.

**Results:**

Of 28 randomized participants, 12 (44.4%) had a treatment-emergent adverse event (TEAE) and 10 (37.0%) had a TEAE considered related to study drug, most of which were mild or moderate. Four participants (18.2%) had urinary TEAEs while receiving danavorexton, all of which were mild in severity. There were no deaths or TEAEs leading to discontinuation. Improvements in MWT, KSS, and PVT scores were observed with danavorexton compared to placebo. Following drug administration, a mean sleep latency of 40 min (maximum value) was observed during the MWT within 2 h of danavorexton infusion in most participants.

**Conclusions:**

A single infusion of danavorexton improves subjective and objective excessive daytime sleepiness in people with IH with no serious TEAEs, indicating orexin-2 receptor agonists are promising treatments for IH.

**Clinical Trial**: Clinicaltrials.gov. https://clinicaltrials.gov/ct2/show/NCT04091438

## Graphical Abstract

**Graphical Abstract:**
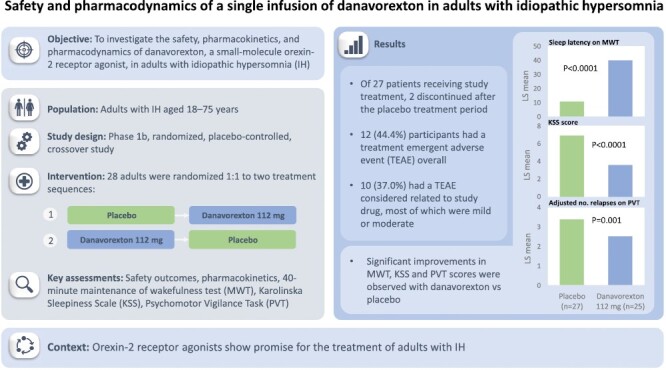

**Statement of Significance**

The pathophysiology of idiopathic hypersomnia is poorly understood, resulting in a lack of specific treatments for the condition. Agents targeting the orexin neuropeptide system have been found to improve wakefulness in people with narcolepsy but have not been investigated in other disorders of hypersomnolence. We found that a single infusion of the orexin-2 receptor agonist danavorexton improves subjective and objective excessive daytime sleepiness in adults with idiopathic hypersomnia, suggesting that orexin-2 receptor agonists hold promise for the treatment of this disabling sleep disorder.

## Introduction

Idiopathic hypersomnia (IH), one of the central hypersomnia sleep-wake disorders, is characterized by chronic excessive daytime sleepiness (EDS) and sleep inertia (awakening that is difficult or accompanied by behavioral abnormalities; also called sleep drunkenness). People with IH may experience additional symptoms, such as prolonged night-time sleep and non-refreshing naps [1, 2], fatigue, cognitive problems, and autonomic dysfunction. Hypersomnolence in IH typically occurs in monotonous, low-engagement situations, but may also occur in higher-engagement activities [3].

Diagnostic criteria for IH, also termed idiopathic CNS hypersomnolence in the International Classification of Sleep Disorders—Edition 3 (ICSD-3) nosology, requires the presence of daytime lapses into sleep or an irrepressible need to sleep present on a daily basis for at least 3 months, the absence of cataplexy, fewer than two sleep-onset rapid eye movement (REM) periods (including nocturnal PSG [nPSG]) on a multiple sleep latency test (MSLT) and either an average sleep latency of ≤ 8 min on the MLST or a total 24-h sleep time of ≥ 660 min. Insufficient sleep syndrome and alternative causes for sleepiness must be excluded [4, 5].

The pathophysiology of IH is unknown. Although orexin (hypocretin), melanin-concentrating hormone, adenosine, acetylcholine, dopamine, norepinephrine, histamine, gamma-aminobutyric acid (GABA), and glutamate are known to be involved in sleep-wake regulation [6], there is no clear evidence for a primary involvement of any of these systems in the pathophysiology of IH [7, 8]. Medications targeting GABA and histamine signaling pathways are used for the treatment of narcolepsy, and agents targeting the orexin system are in development; however, few clinical studies have been undertaken in populations with IH.

Two orexin neuropeptides, orexin A and orexin B (also called hypocretin-1 and hypocretin-2) act through the G-protein coupled orexin-1 and orexin-2 receptors (OX1R and OX2R). In normal sleep-wake regulation, orexin is believed to play a key role in arousal/wakefulness stabilization [9, 10], stabilization of sleep inertia [11], REM sleep regulation, and stabilization of the REM sleep muscle atonia circuit—the driver of cataplexy in people with narcolepsy type 1 (NT1) [7, 10]. Low cerebrospinal fluid (CSF) levels of orexin are associated with NT1 [12, 13], and orexin peptide replacement promoted wakefulness in animal models of NT1 [14]. Although orexins are present in the CSF at normal concentrations in other hypersomnolence disorders, including IH [12, 15], findings from preclinical and clinical studies suggest that OX2R agonists may be useful for treating populations with primary sleep disorders not associated with orexin deficiency. In preclinical studies, non-peptide OX2R-selective agonists promoted wakefulness both in mouse models of narcolepsy and in wild-type mice [16, 17]. In clinical studies, danavorexton, a small-molecule OX2R-selective agonist [17, 18], was found to increase wakefulness in people with narcolepsy type 2 (NT2) [19] and in people with obstructive sleep apnea who experienced residual sleepiness despite treatment [20].

The aim of this study was to evaluate the safety, pharmacokinetics, and pharmacodynamics of danavorexton administered to adults with IH.

## Methods

### Study design

This was a phase 1b, randomized, double-blind, placebo-controlled, two-period, two-treatment crossover study of a single IV infusion of danavorexton in adults with IH. The study was conducted between January 2020 and November 2020 in the United States and Japan (NCT04091438), and consisted of a 28-day screening period, two 1-day treatment periods separated by a 1-day washout period, and a 1-week follow-up period. A 1-day washout period was selected for this study based on pharmacokinetic findings from previous research, which indicated a short half-life and return of pharmacokinetic and physiologic responses to pre-dose levels within 24 h [21]. The study was conducted according to the International Conference on Harmonization of Good Clinical Practice guidelines and the principles of the Declaration of Helsinki, and in compliance with Institutional Review Board regulations and all applicable local regulations. A manually signed and dated informed consent form and any required privacy authorization was required for each participant.

### Participants

Individuals meeting the following criteria were eligible for inclusion: aged 18–75 years inclusive; IH diagnosed according to ICSD-3 criteria, verified by nPSG and the MSLT within the prior 10 years; 7-day mean nightly sleep duration of ≥ 420 min (as measured by 7 days of actigraphy and sleep diary); mean sleep latency (of four sessions) on the maintenance of wakefulness test (MWT) of ≤ 20 min at baseline with no single MWT session having a sleep latency of > 30 min; and an Epworth Sleepiness Scale (ESS) score of ≥ 11 at screening and on study day −2.

Key exclusion criteria included a usual bedtime later than midnight or an occupation requiring night-time or variable shift work; a history of a sleep disorder other than IH; or a health condition that could affect the safety of the participant, including coronary artery disease, prior myocardial infarction, angina, cardiac rhythm abnormality, heart failure, cardiac ischemia, epilepsy, or seizures, or any unstable behavioral or psychiatric disorder, including major depression or active suicidal ideation. Tobacco and nicotine-containing products, caffeine, and alcohol were prohibited during the study confinement periods. Concomitant medications, including psychostimulants, antipsychotic drugs, mood stabilizers, muscle relaxants, moderate or potent CYP3A inhibitors and inducers, and any sedating agents, were prohibited from the longer of 7 days or five half-lives before the administration of study drug. Sodium oxybate was required to be discontinued at least 4 weeks before screening.

Following screening, participants underwent nPSG to confirm the absence of other comorbid sleep disorders and were required to have an apnea-hypopnea index of ≤ 10/h (defined according to the American Academy of Sleep Medicine recommended scoring for obstructive hypopneas [22]) and a periodic limb movement arousal index of ≤ 15/h.

### Treatment

**Figure 1.**
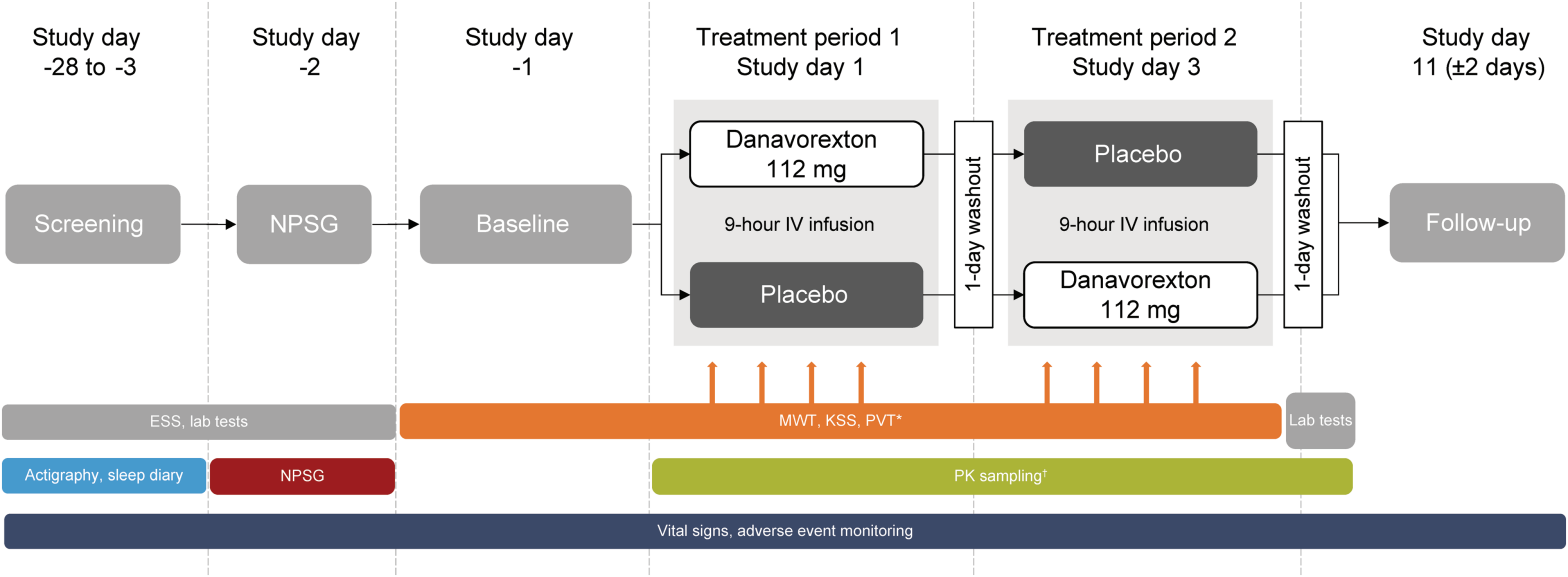
Study design. *During each treatment period, four 40-min MWT sessions were performed at 2, 4, 6, and 8 h after the start of infusion. Each MWT session was followed by administration of the KSS and then the PVT. †During each treatment period, blood for PK analysis of danavorexton (and its metabolites) was obtained before infusion, at 1, 3, 5, 7, and 9 h after start of infusion, and at 0.17, 1, 4, and 15 h after the end of the infusion on day 1. The 15-h post-dose assessment was on day 2. ESS, Epworth Sleepiness Scale; IV, intravenous; KSS, Karolinska Sleepiness Scale; MWT, maintenance of wakefulness test; nPSG, nocturnal polysomnography; PK, pharmacokinetic; PVT, psychomotor vigilance task.

Participants were randomized 1:1 to one of two treatment sequences using the interactive web response system (Figure 1). After randomization, participants received danavorexton 112 mg (16.67% solution in 0.09% sodium chloride) and placebo (0.09% sodium chloride) in the order defined by the sequence to which they were randomized. On day 1 of each treatment period, a total volume of 675 mL danavorexton or placebo was administered via a single 9-h intravenous (IV) infusion at a rate of 75 mL/h, commencing at approximately 08:00.

### Assessments

Primary endpoints were percentage of participants with ≥ 1 treatment-emergent adverse event (TEAE) and the percentage of participants meeting markedly abnormal criteria for clinical safety laboratory tests, vital signs, or 12-lead electrocardiography (ECG) at least once. Safety assessments included full physical examinations, vital signs, and 12-lead ECG—performed at screening, baseline, and during each treatment period—and monitoring of adverse events (AEs) throughout the study. AEs were coded by system organ class and preferred term using the Medical Dictionary for Regulatory Activities (MedDRA) version 23.0. A TEAE was defined for each treatment period as an AE with onset on or after the first dose of the study drug and before the first dose of study drug in the next treatment period. Pharmacokinetics were evaluated in all participants who received danavorexton; to do so, serial blood samples were collected before infusion and up to 24 h after infusion start.

Exploratory endpoints included the effects of danavorexton on EDS as measured using the 40-min MWT [23] and the Karolinska Sleepiness Scale (KSS) [24], and the effects of danavorexton on attention and vigilance, using the 10-min psychomotor vigilance task (PVT). The 40-min MWT measures a person’s ability to stay awake under soporific conditions. Four 40-min MWT sessions were performed at 2, 4, 6, and 8 h post-infusion start, during which participants were instructed to stay awake. Sleep latency was measured using a standard electroencephalogram (EEG), electromyogram (EMG), and electrooculogram (EOG) montage. Latency to sleep onset was recorded for each session and averaged to derive a mean sleep latency. After each MWT session, participants completed the KSS and then the PVT (Figure 1). The KSS is a 10-point self-rating scale measuring subjective sleepiness (1 = Extremely alert to 10 = Extremely sleepy, can not keep awake). The PVT is a 10-min task in which participants are instructed to press a button as soon as possible after a visual stimulus appears on the screen. PVT endpoint measurements included adjusted number of lapses (sqrt[number of lapses] + sqrt[1 + number of lapses]) and reaction time [RT] ≥ 500 ms in each trial.

### Statistical analyses

The statistical significance of the MWT, KSS, and PVT endpoints was evaluated using a linear mixed effect model with effects of treatment, period, sequence, and participant within sequence. The least squares (LS) mean over the four post-dose time points and associated standard error (SE) and 95% confidence intervals (CIs) for each treatment were estimated along with all pairwise differences from placebo and associated SEs, 95% CIs, and *p*-values. Up to 40 participants were planned to be randomized equally to each treatment sequence to ensure 36 participants completed the study (assuming a 10% dropout rate). An interim analysis was planned to occur when at least 12 participants had completed both treatment periods.

## Results

### Participant disposition and baseline characteristics

**Figure 2.**
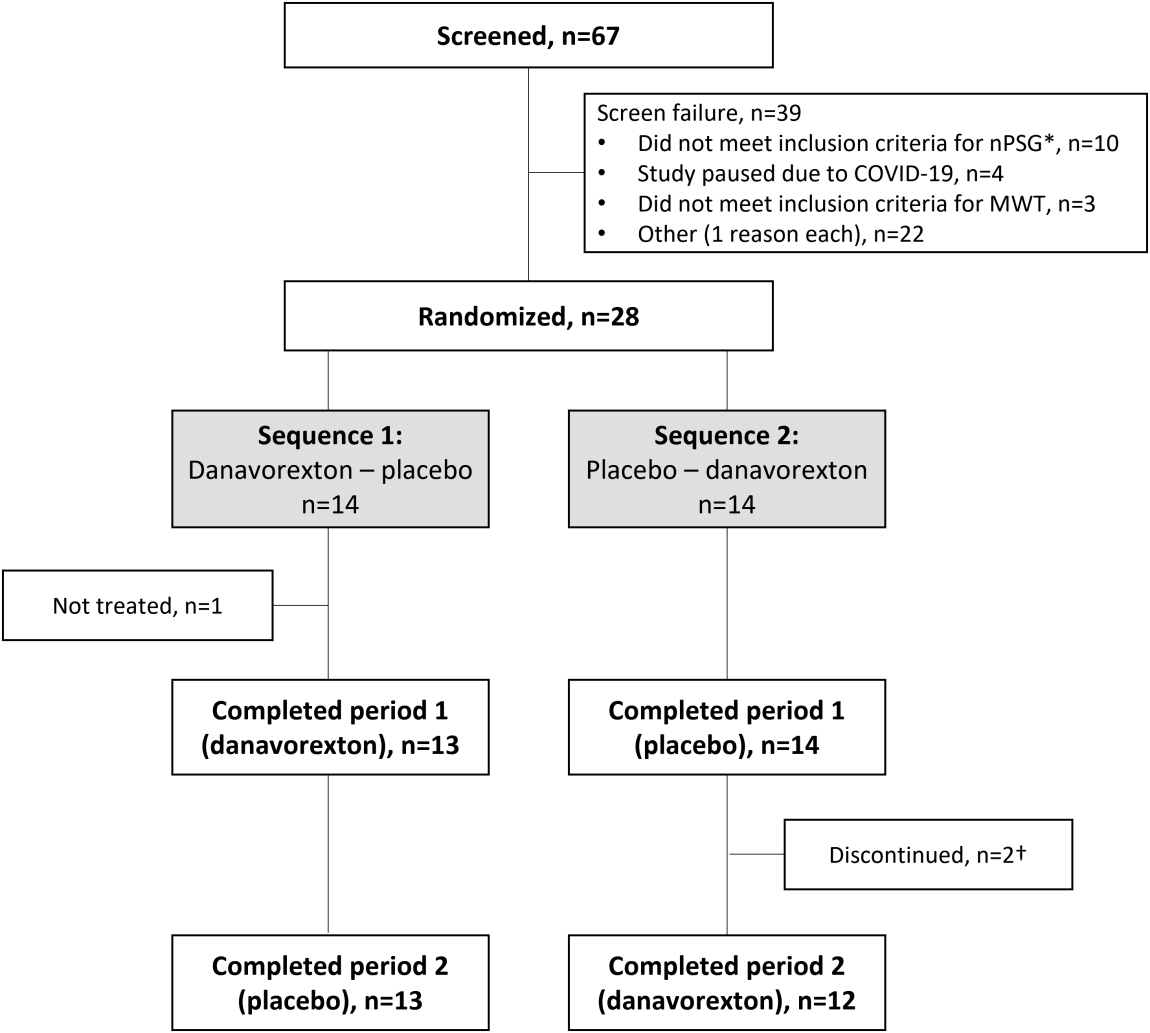
Participant disposition. *nPSG inclusion criteria, evaluated at study day −2, demonstrated that subjects had no other comorbid sleep disorders or clinically significant nocturnal hypoxemia (O_2_ saturation ≤ 80% for ≥ 5% of total sleep time), AHI was ≤ 10/h, PLMAI was ≤ 15/h, and total sleep time ≥ 6.5 h. †Two participants discontinued the study after receiving placebo and never received danavorexton.

A total of 28 participants meeting criteria for IH (as described earlier) were enrolled and randomized. The stopping criteria based on changes in sleep latency in the MWT were met in the planned interim analysis and further recruitment into the study was terminated early. One participant was randomized but not treated owing to difficulties inserting the infusion line. Of 27 participants receiving treatment, two discontinued the study after receiving placebo and never received danavorexton; one was randomized in error, despite not meeting PSG criteria, and another was accidentally registered as an early termination by the study site (Figure 2). Both patients were included in placebo-group safety and efficacy analyses. Based on 7 days of actigraphy data, the mean total sleep time per 24-h period was 651 min, with 54% of participants having a total sleep time of ≥ 660 min. Most participants were female (78%) and white (67%). Baseline characteristics are summarized in Table 1.

**Table 1.** Baseline characteristics

Parameter	Total
Age, years	*n* = 27
Mean (SD)	31.7 (8.79)
Median (min, max)	31.0 (19, 52)
Sex, *n* (%)	*n* = 27
Male	6 (22.2)
Female	21 (77.8)
Race, *n* (%)	*n* = 27
Asian (Japanese)	4 (14.8)
Black or African American	4 (14.8)
White	18 (66.7)
Not reported	1 (3.7)
BMI, kg/m^2^	*n* = 27
Mean (SD)	25.3 (3.86)
Median (min, max)	25.8 (18.3, 32.6)
Sleep latency on MWT, minutes	*n* = 26
Mean (SD)	9.2 (5.49)
Median (min, max)	9.7 (1.1, 19.4)
Estimated total sleep time, minutes^*^	*n* = 26
Mean (SD)	650.8 (96.75)
Median (min, max)	668.9 (459.4, 928.7)
ESS score	*n* = 27
Mean (SD)	18.3 (3.34)
Median (min, max)	18.0 (12, 24)
KSS score	*n* = 27
Mean (SD)	7.3 (1.8)
Median (min, max)	8.0 (3.0, 9.0)

BMI, body mass index; ESS, Epworth Sleepiness Scale; KSS, Karolinska Sleepiness Scale; MWT, maintenance of wakefulness test; SD, standard deviation.

^*^Total sleep time calculated over 7 days of actigraphy, performed from days −9 to −3 prior to baseline.

### Safety analyses

Overall, 12 participants (44.4%) had TEAEs, 10 (37.0%) of which had TEAEs considered related to study drug. Most TEAEs were mild or moderate, with one case of severe insomnia during the danavorexton 112 mg treatment period. No deaths, SAEs, or TEAEs leading to discontinuation occurred during the study. Four participants (16.0%) had urinary TEAEs during the danavorexton treatment period (pollakiuria in three participants, and micturition urgency, polyuria, and increased urinary output in one participant each), all of which were mild in severity. No urinary-related TEAEs occurred during the placebo treatment period. The most common TEAEs during the danavorexton treatment period were pollakiuria, dizziness, rhinorrhea, and headache (Table 2). Dry mouth, salivary hypersecretion, feeling jittery, urine output increased, anxiety, euphoric mood, micturition urgency, polyuria, sneezing, insomnia at initiation, and persistent insomnia occurred in one participant each during the danavorexton treatment period. Two participants reported headache and one reported administration site phlebitis during the placebo treatment period.

**Table 2.** Summary of TEAEs

	Placebo (*n* = 27) *n* (%)	Danavorexton (*n* = 25) *n* (%)	Total (*n* = 27) *n* (%)
Any TEAEs	4 (14.8)	10 (40.0)	12 (44.4)^*^
Mild	3 (11.1)	10 (40.0)	12 (44.4)^*^
Moderate	1 (3.7)	1 (4.0)	2 (7.4)
Severe	0	1 (4.0)	1 (3.7)
Treatment related	2 (7.4)	9 (36.0)	10 (37.0)^*^
Serious TEAEs or deaths	0	0	0
TEAEs > 1 individual			
Headache	2 (7.4)	1 (4.0)	3 (11.1)
Pollakiuria	0	3 (12.0)	3 (11.1)
Dizziness	0	2 (8.0)	2 (7.4)
Rhinorrhea	0	2 (8.0)	2 (7.4)

TEAE, treatment-emergent adverse event.

^*^Individual participants may have experienced TEAEs in both the placebo and danavorexton treatment periods.

No clinically significant changes in serum chemistry, hematology (hematocrit, hemoglobin, platelets, red/white blood cell counts), urinalysis, heart rate measurements, or ECG (mean heart rate or QTcF interval values) were reported in comparison with baseline. On 12-lead ECG, one participant had a marked increase in PR interval of ≥ 200 ms following placebo administration, and six had a reduction in QRS duration to ≤ 80 msc (three while receiving placebo and three while receiving danavorexton).Overall, mean BP was elevated with danavorexton compared with placebo during infusion (SBP: LS mean increase of 3.99 mmHg vs placebo; DBP: LS mean increase of 3.58 mmHg vs placebo), with no clinically relevant differences between treatment groups 24 h post-infusion start. Markedly abnormal blood pressure values were observed in both treatment periods; all those occurring while on danavorexton were low. Four participants had markedly abnormal diastolic blood pressure (DBP; <50 or ≥ 100 mmHg or > 20 mmHg change from pre-dose) values at one or more time points: one (4.0%) had an abnormal DBP value (<50 mmHg) while receiving danavorexton, and four (14.8%) had a change from the pre-dose value of > 20 mmHg while receiving placebo. Eight participants had markedly abnormal systolic blood pressure (SBP; <90 or ≥ 160 mmHg or > 20 mmHg change) values at one or more time points: SBP < 90 mmHg was observed in three participants (12.0%) while on danavorexton, and in four participants (14.8%) while on placebo; and SBP change from pre-dose > 20 mmHg was observed in three (11.1%) participants receiving placebo. Two of the participants had SBP < 90 mmHg following both placebo and danavorexton treatment periods.

### Pharmacokinetics

Danavorexton pharmacokinetics were consistent with previously published findings [21]. Following a single 9-h IV infusion, the time for danavorexton plasma concentration to plateau ranged between approximately 3 and 7 h after the start of infusion. Mean plasma concentration declined rapidly following the end of infusion, with an estimated mean half-life of 3.05 h, and remained above the limit of quantification at 24 h post-infusion start.

### MWT

**Figure 3.**
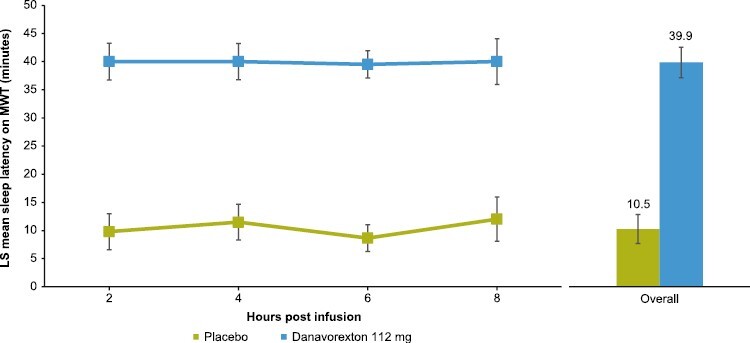
Effect of danavorexton on sleep latency on the MWT (minutes). LS, least squares; MWT, maintenance of wakefulness test.

At baseline, mean sleep latency on the MWT was 9.2 min. Post-infusion start, sleep latency on the MWT was significantly higher after danavorexton vs placebo at all time points (*p* < 0.001; Figure 3). Average sleep latency values were 10.5 and 39.9 min for placebo and danavorexton, respectively. LS mean (95% CI) placebo-adjusted sleep latency over the four MWT sessions was 29.4 (25.64, 33.17) with danavorexton (*p* < 0.0001). With danavorexton, 24 of 25 participants (96%) reached the maximum sleep latency of 40 min in at least one of the four sessions, compared with only one of 27 participants (3.8%) receiving placebo.

### KSS

**Figure 4.**
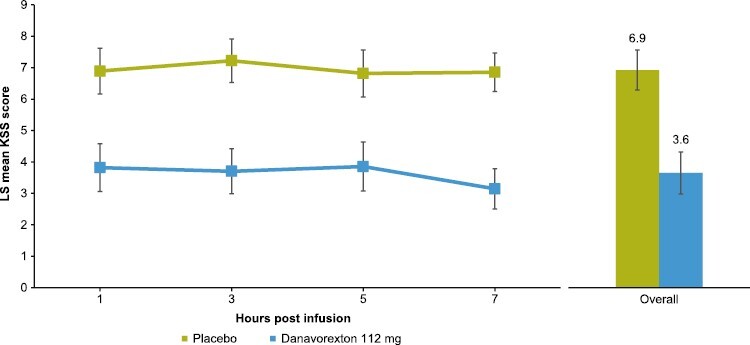
Effect of danavorexton on KSS score. KSS, Karolinska Sleepiness Scale; LS, least squares.

KSS scores were significantly lower after danavorexton vs placebo at all time points, indicating reduced subjective sleepiness (*p* < 0.001; Figure 4). The LS mean KSS scores over the four time points were 6.95 and 3.63 for placebo and danavorexton, respectively, and the LS mean (95% CI) placebo-adjusted KSS score over the four time points was −3.31 (−4.15, −2.48) with danavorexton (*p* < 0.0001).

### PVT

Significant improvements in sustained attention/vigilance, as assessed by the PVT, were observed after danavorexton. For each endpoint, the values were averaged over the four time points post-infusion start. LS mean values for adjusted number of lapses were 3.38 and 2.52 for placebo and danavorexton, respectively, and the LS mean placebo-adjusted difference was −0.85 (95% CI: −1.3, −0.4; *p* = 0.001). For RT (reaction time), LS mean values were 307.82 and 282.65 ms for placebo and danavorexton, respectively, reduced from 360.45 and 308.35 ms at baseline, and the placebo-adjusted LS mean difference was −25.17 ms (95% CI: −38.05, −12.29; *p* < 0.001). For mean reciprocal RT, LS mean values were 3.55 and 3.77 1/s for placebo and danavorexton, respectively, and the LS mean placebo-adjusted difference was 0.22 1/s (95% CI: 0.1, 0.34; *p* = 0.001) for danavorexton compared with placebo. On the PVT, mean reaction times and number of lapses were reduced during danavorexton administration compared to placebo.

## Discussion

The orexin system plays an important role in the regulation of wakefulness, and although CSF orexin neuropeptide levels are normal in most people with IH [15], variants associated with the dysfunction of orexin signaling have been identified in people with IH and self-reported EDS [25, 26]. Further, previous studies have shown that activation of OX2R within the orexin system increases wakefulness in wild-type mice [17], as well as in people with NT2 [27] or sleep apnea [20] with normal orexin concentrations, suggesting these drugs are effective even when orexin system neurons are not damaged. In this phase 1b crossover study, we demonstrated that a single dose of danavorexton, a selective orexin-2 agonist, was generally well tolerated and improved EDS and measures of attention/vigilance in adults with IH.

Overall, there were no serious TEAEs, no discontinuations due to TEAEs, no markedly abnormal laboratory values, and few markedly abnormal vital signs associated with danavorexton administration, consistent with a previous evaluation of danavorexton in a similar study involving people with obstructive sleep apnea [20]. A similar proportion of participants reported urinary TEAEs while receiving danavorexton in both studies, with pollakiuria being the most frequently reported urinary AE in both. Increases in blood pressure observed after danavorexton administration were of a similar magnitude across the two studies and returned to pre-dose values within 24 h of infusion start. These findings are consistent with preclinical studies, in which orexin A in the spinal cord of normal rats was found to activate the micturition reflex, although via OXR1 [28], and with observations showing that central injection of orexin A affects renal sympathetic nerve activity and blood pressure in rats [29, 30]. Additional studies will be needed to determine if OX2R is also involved in micturition, either alone or upstream of the OXR1 response.

We found that a single, constant infusion of danavorexton 112 mg over 9 h resulted in improvements in objective (MWT) and subjective (KSS) measures of wakefulness and improvements in sustained attention/vigilance (PVT) in people with IH. In our study, most participants receiving danavorexton remained awake for the maximum time of 40 min from the first MWT session at 2 h post-infusion start and in all subsequent MWT sessions over the infusion period, achieving sleep latency times comparable to or higher than those of healthy participants [31]. These findings represent considerable and rapid improvements in sleep latency of approximately 30 min from baseline within 2 h of danavorexton 112 mg infusion start that were not observed with placebo and suggest that evaluation of lower doses may be warranted in future studies of people with IH. In comparison, small improvements in sleep latency were achieved with oral modafinil, a wake-promoting agent that exerts its effects through dopaminergic reuptake inhibition [32, 33]. At a dose of 200 mg once daily, oral modafinil resulted in sleep latency improvements of ~3 min after 3 weeks in people with IH [34].

Participants receiving danavorexton reported levels of sleepiness on the KSS that were comparable with the general population. In addition, improvements in sustained attention/vigilance were observed using the PVT, a simple RT test, after initiation of the danavorexton infusion, despite the fact that most participants did not present with a clinically relevant impairment in vigilance with this test at baseline (clinical impairment defined as mean RT ≥ 500 ms [35]). With danavorexton, mean RT values and reciprocal RT values were close to those from healthy controls in a previous study [36]. Both the KSS and PVT are most frequently utilized in the evaluation of sleep-deprived but otherwise healthy individuals, although the PVT has been proposed as a potential diagnostic tool for IH [36]. These findings warrant further investigation of the PVT in larger populations of people with IH, including those who have severely impaired vigilance at baseline.

Limitations of the study include a short treatment duration and small participant numbers that, although typical for phase 1b studies, limits the generalizability of our findings to other populations outside the United States and Japan and to non-inpatient settings. Of note, findings were not evaluated for participants from the United States and Japan separately, and thus geographic differences have not been determined. Furthermore, the standard nPSG performed does not allow the identification of long sleepers, as the mean total sleep time on the standard 8-h nPSG was 7.1 h. Finally, the literature is consistent in stating an accurate and reproducible diagnosis of IH and differentiation from NT2 is difficult. Taking that into consideration, we utilized the only presently validated criteria for the diagnosis of IH; that is, we used the ICSD-3 criteria for categorization of IH, using the results of PSG/MSLT data provided by individual investigators, showing a mean sleep onset latency of < 8 min over 5 naps and the presence of ≤ 1 sleep onset REM period (SOREMP), along with MWT data showing a sleep latency of < 20 min.

Additional studies with other orexin agonists in larger samples of IH patients are needed to extend on these promising findings. Further, research aimed at better understanding the role or impact of the orexin system in the pathophysiology of IH is also needed.

## Data Availability

The datasets, including the redacted study protocol, redacted statistical analysis plan, and individual participants’ data supporting the results of the study, will be made available after the publication of study results within 3 months from initial request to researchers who provide a methodologically sound proposal. The data will be provided after its de-identification, in compliance with applicable privacy laws, data protection, and requirements for consent and anonymization.
